# P54 Fidaxomicin EXTEND for treating first occurrence of *Clostridioides difficile* infection in the adult haematology population

**DOI:** 10.1093/jacamr/dlag102.060

**Published:** 2026-06-26

**Authors:** Ahmad B Simreekheea, Unell B G Riley, Stephanie J Smith

**Affiliations:** The Royal Marsden NHS Foundation Trust, London, UK; The Royal Marsden NHS Foundation Trust, London, UK; The Royal Marsden NHS Foundation Trust, London, UK

## Abstract

**Background:**

*Clostridioides difficile* (CDI) is a gastrointestinal infection associated with significant morbidity and mortality. Haemato-oncology patients are at higher risk due to long admissions, immunosuppression and frequent antibiotic use. Fidaxomicin, a macrocyclic antibiotic, offers selective antibacterial action against *C. difficile*. It is the recommended first-line therapy according to ESCMID compared to NICE, as it preserves healthy gut flora and prevents recurrence. Vancomycin-resistant *Enterococcus faecium* (VRE) is an opportunistic pathogen monitored in immunocompromised patients and has been strongly linked with CDI rates.

**Objectives:**

To assess the clinical response to Fidaxomicin EXTEND as first-line therapy, the relapse rate and prevention of VRE in haemato-oncology patients with CDI.

**Patients and methods:**

All Adult Haematology patients (≥18-years-old) with clinical symptoms of CDI were tested for glutamate dehydrogenase (GDH) and toxin A & B by C.DIFF QUIK-CHEK COMPLETE^®^. Project exclusion criteria included: Patients <18years-old/ under the paediatric team, those unable to tolerate oral treatment, or those already prescribed concurrent CDI antibiotics (i.e. Metronidazole). Patient demographics, underlying cancer diagnosis, CDI risk factors, length of stay (LOS) and relapse rate up to 3 months since treatment were collected. Stool type and frequency were reviewed at 5 day time-points up to day 35 (D0-D35). Data was prospectively collected and compared to 2024 when oral vancomycin was the first-line agent, utilizing the EPIC electronic prescribing system.

**Results:**

A total of 7 patients out of 8 were started on Fidaxomicin EXTEND between 02/2025 and 07/2025. The most prevalent haematological malignancy with CDI was leukaemia (72% *n*=5. Of the allogeneic transplant patients (43%), only 1 patient developed confirmed GvHD during their CDI. All patients had more than 3 risk factors for CDI, the most prevalent being antibiotic use and receiving chemotherapy during their admission or within 1 month before CDI (71%). Of the patients treated with Fidaxomicin, 72%(*n*=5) of non-elective patients in 2025 had a length of stay (LOS) of 9 days during this treatment, versus 64% (*n*=7) in 2024 with a LOS of 29.5 days. Patients treated with Fidaxomicin EXTEND had a quicker resolution of their diarrhoea and return to their baseline compared to vancomycin. In 2025, 1 patient (14%) had recurrence within 3 months on Fidaxomicin EXTEND versus 3 patients (30%) in 2024 receiving vancomycin. Equally, 3/7 patients acquired VRE with fidaxomicin (1 developed after 4 months) compared to 2024, where 7/11 acquired VRE (2 developed within 4 months) on oral vancomycin.

**Conclusions:**

Although this is a small number of patients and the study is ongoing, it suggests Fidaxomicin EXTEND offers significant advantages in the management of CDI in adult haematology patients. These include improved cure rates, reduced recurrence, and lower rates of VRE colonization. Its adoption may inform broader quality improvement initiatives across hospital Trusts and similar healthcare environments in managing these complex oncology groups. However, further data and expanded trials are needed to confirm if this trend is significant, which could improve the quality of care in this high-risk patient population.

